# Circulation Fluidized Bed Combustion Fly Ash as Partial Replacement of Fine Aggregates in Roller Compacted Concrete

**DOI:** 10.3390/ma12244204

**Published:** 2019-12-14

**Authors:** Wei-Ting Lin, Kae-Long Lin, Kailun Chen, Kinga Korniejenko, Marek Hebda, Michał Łach

**Affiliations:** 1 Department of Civil Engineering, National Ilan University, No.1, Sec. 1, Shennong Rd., I-Lan 260, Taiwan; 2 Department of Environmental Engineering, National Ilan University, No.1, Sec. 1, Shennong Rd., I-Lan 260, Taiwan; kllin@niu.edu.tw; 3 School of Environment and Architecture, University of Shanghai for Science and Technology, 516 Jungong Road, Yangpu District, Shanghai 200093, China; chenkailun21@gmail.com; 4 Institute of Materials Engineering, Faculty of Materials Engineering and Physics, Cracow University of Technology, Warszawska 24, 31-155 Kraków, Poland; kkorniej@gmail.com (K.K.); mhebda@pk.edu.pl (M.H.); mlach@pk.edu.pl (M.Ł.)

**Keywords:** Circulating fluidized bed combustion technology, SEM, XRD, sulfate resistance, green materials

## Abstract

Recently, many people around the world have been concerned with environmental protection and sustainability. The goal of various countries’ research has been focused on how to regenerate existing resources. Circulation fluidized bed combustion (CFBC) technology is one of the emerging combustion technologies for electricity generation and produces more than 800,000 tons of CFBC fly ash (CFA) per year for combustion. CFA has been widely applied in cement additive, new building materials and cement-based materials. The goal of this study was to discuss the engineering properties of roller-compacted concrete containing CFA. Test subjects included compressive strength, flexural strength, absorption, setting time, unit weight, sulfate resistance, SEM microscopic observations and XRD ingredient analysis. Test results indicate the following: (1) using CFA as a substitute of fine aggregates up to 10 wt.% would improve the development of later flexural strength; (2) the increases in pre-pressure would increase the compressive strength and unit weight and decrease absorption; (3) using CFA would reduce the initial setting time by 30%–60% and reduce the final setting time by 16%–20%; (4) using CFA would reduce the absorption; (5) using CFA would reduce the unit weight by 0.5%–2.8%, and the increases in pre-pressure would increase the unit weight by about 0.9%–2.1%; (6) CaO in CFA helps to improve sulfate resistance; (7) scanning electron microscopy (SEM) observation shows that the increases in pre-pressure would reduce the pores; and (8) X-ray diffraction (XRD) analysis shows that the inclusion of CFA would increase the content of Ca(OH)_2_ in concrete.

## 1. Introduction

Since the Industrial Revolution in the 19th century, the exhaustion of resources has seemed to be an insoluble problem. With the rapid development of science and technology, the value of non-renewable resources has soared, and industrial waste has become a serious problem. In view of the above, the development of new resources and the reuse of wastes have become crucial issues for achieving sustainable developments in the 21st century. Circulating fluidized bed combustion technology (CFBC) has become one of the most widely applied combustion technologies in recent years [1,2]. Compared to conventional pulverized coal combustion technology, CFBC presents greater performance and lower pollutant emissions. Furthermore, it is suitable for a variety of fuels, and its application to petroleum coke with a sulfur content of less than 5% firmly follows the principle of waste recycling [3]. However, the widespread use of CFBC has also led to an annual increase in the production of CFBC fly ash (CFA), which requires additional methods for reuse or disposal. Several research works have been conducted on the use of CFA in cement manufacturing, soil improvement, road basements, new building materials and building fillers [1,4]. In Taiwan, CFA has been applied to cement-based materials [1,2,5,6]. The substitutes in construction materials are often adopted to reduce the use of raw materials. However, by-products such as fly ash are usually accompanied as aggregates or cement substitutes due to industrial development [7,8].

Roller-compacted concrete (RCC) is a type of no-slump concrete which is easy to use and economical [9,10]. RCC is extremely suitable for regions in which rapid construction is required—especially in Taiwan, where rain and moisture often extend construction time. Compared to traditional ordinary concrete, RCC contains more coarse aggregates and less cement per unit volume, and the fresh RCC must be able to support the vibratory roller [10,11]. Previous research has determined that the application of fly ash, slag or pozzolan to RCC can enhance its engineering properties, such as its compressive strength and flexural strength [7,12,13,14]. With the application of RCC in dam construction, which began in the 1970s, RCC has gradually received increasing attention in other engineering applications. For example, its use on road pavements is known as roller-compacted concrete pavements (RCCP) and it is commonly used in timber storage warehouses, parking lots, storage areas, military roads and hydraulic structures [15]. Research and development worldwide has matured RCCP techniques. In heavy traffic loads, the traditional concrete pavements have a short service life and require frequent maintenance. The rutting problems of asphalt concrete pavements are a focal problem. Therefore, engineers are paying increased attention to the development of RCCP, which has lower development costs and good durability [15,16].

Mineral admixtures are common materials for concrete, including fly ash, slag, and silica fume. They are mainly used as substitute binders to improve workability and durability [4,17]. They react chemically with calcium hydroxide at room temperature to form compounds with cement properties in the presence of moisture [18]. Based on the calcium hydroxide produced by the hydration of cement and amorphous siliceous or aluminous materials, the chemical reaction is referred to as a Pozzolanic reaction or secondary hydration reaction. The results include C–S–H or C–A–S–H gel which enhances the mechanical properties and durability of the mixture and increases the compactness of the interior structure of the concrete. Fly ash is a common pozzolanic material in RCC. Due to the fine spherical shape of fly ash particles, the addition of fly ash to cementitious materials helps in the improvement of characteristics such as their workability and later strength. Cao et al. [19] studied the replacement of cement with high-volume fly ash in RCC. The substitution ratio in the specimens was from 45% to 95% and the compression was performed at 50 g/cm^2^ for 120 s. The results revealed that the specimens containing fly ash had 50% greater strength than specimens without fly ash at the age of 90 days. Atiş observed that the inclusion of high amounts of fly ash in RCC attained higher compressive and flexural strength properties compared to original Portland concrete and that it can serve as an alternative material for road pavement [20]. Sun examined the fatigue strength of 100 × 100 × 400 mm^3^ RCC specimens compacted at 50 g/cm^2^ for 120 s [21]. Fly ash replaced 0% to 45% of cement, and the results indicated that the addition of fly ash can increase the fatigue strength by 40% to 50%. Atiş [20] indicated that non-standard high-CaO (61.9%) fly ash replacing 0% to 45% of cement can increase early compressive strength (3 days). At 28 days, the specimens containing 15% fly ash presented a higher compressive strength than specimens without fly ash. The specimens containing 30% fly ash required 91 days to develop greater compressive strength than specimens without fly ash.

CFA is one of the by-products of the newly developing CFBC technology. Due to its high calcium sulfate (CaSO_4_) content, it has a quick hydration reaction and then maintains good cementitious properties [1]. Chi [16] indicated that the use of CFA instead of fine aggregates favors the strength development of RCC. However, the maximum replacement is 15%. Increasing the pre-pressure reduces water absorption and the initial surface absorption and increases the compressive strength, splitting strength, and durability. Chi investigated only a pre-pressure ranging from 25 g/cm^2^ to 75 g/cm^2^. Depending on the weight percentage of fine aggregate substitutes, CFA has a positive influence on strength development. Furthermore, the sulfate resistance test revealed that dihydrated calcium sulfate is formed in the specimens, thereby increasing the compactness. The compressive strength of specimens prepared with a paste of 35% water and 65% CFA was tested by Conn and Sellakumar et al. [22]. The results indicated that the specimens have good cementitious properties due to the high CaSO_4_ content, and the authors pointed out that the strength of the CFA is derived from the reaction of CaO and CaSO_4_. Bland et al. [23] applied CFA to RCC; they also found that it can enhance strength development [24]. Few studies have reported using CFA as a partial replacement of fine aggregates under various roller compaction pressures in RCC, and this seems to be a suitable solution for one kind of circular economy. Administering a suitable dosage for applications with RCC is also an important task. The objective of this study was to apply CFA to RCC, increase the weight percentage of fine aggregate substitutes to 30%, and adopt a pre-pressure ranging from 50 g/cm^2^ to 150 g/cm^2^. This study also aims to investigate the combined influence of various replacement amounts and pre-pressure on RCC engineering performance, including compressive strength, flexural strength, water absorption, setting time, unit weight, sulfate resistance, SEM observations, and XRD spectra. Therefore, the results can verify whether CFA is suitable for RCC as a partial replacement of fine aggregates.

## 2. Materials and Methods

### 2.1. Materials

In this study, we used ASTM Type I ordinary Portland cement manufactured from the Asia Cement Corporation (Taipei, Taiwan). The coarse aggregates comprised graded gravel along the Lanyang River in Yilan County, with a specific gravity of 2.64 and absorption of 1.14% under saturated surface dry conditions (SSD). The fine aggregates were from natural river sand with an SSD-specific gravity of 2.57 and absorption of 1.43%.

**Figure 1:**
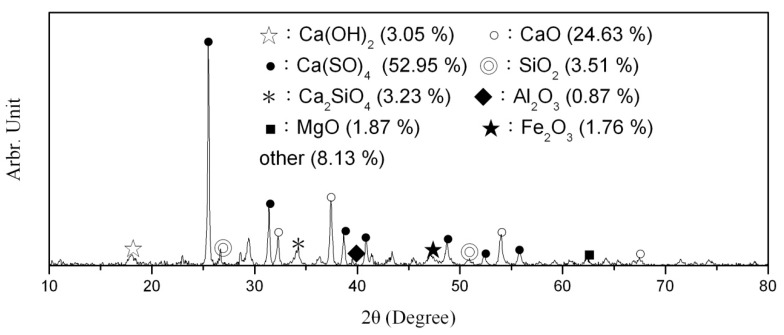
X-ray diffraction (XRD) pattern of circulation fluidized bed combustion (CFBC) fly ash (CFA).

The CFA, a gray–white powder, was produced by the Formosa Plastic Group’s sixth naphtha cracker at Mailiao (City, Country) and came from the CFBC boilers cofiring coal and petroleum coke. SEM observations of the CFA revealed irregular polygonal particles similar to those of slag and rough surfaces. The specific gravity of the CFA is in the range from 2.50 to 2.70. The particles passing through a No. 200 sieve (75 μm) make up roughly 86% of the total, and the fineness is between 2884 cm^2^/g and 3051 cm^2^/g. The primary chemical constituents of the CFA included calcium oxide (CaO) and sulfur trioxide (SO_3_) as well as less silica (SiO_2_). The mineral phases included CaSO_4_ (anhydrite) and CaO (lime), as shown in Figure 1 [2], and among heavy metals, only the nickel (Ni) content was close to the control standard established by the Environmental Protection Administration, as shown in Table 1. Table 1 and Table 2 present the results of the elemental analysis using inductively coupled plasma mass spectrometry (ICP-MS) and chemical analysis using energy-dispersive X-ray spectroscopy (EDS). The standard deviation of EDS analysis can be controlled to less than 3%, which is in agreement with the results of a previous study [25].

### 2.2. Mix Design and Test Methods

The activity index (43%) of CFA is lower than that required for pozzolanic materials (75% according to ASTM C311) [2], so it is less suitable as a substitute binder. Therefore, the selected replacement levels of CFA were 0%, 10%, 20% and 30% by weight of fine aggregates, and the applied levels of pre-pressure were 50 g/cm^2^, 100 g/cm^2^, and 150 g/cm^2^. For example, the pre-pressure was applied on the cylindrical specimens of φ 100 mm × 200 mm using a loading of 4 kg in a circular area of 78.5 cm^2^. Table 3 presents the mix designs of the various specimens in accordance with the recommendation in ACI 211.3R. The target of the designed compressive strength in control specimens was 35.0 MPa at the age of 28 days.

The numbering system was as follows: the first numbers indicate the pre-pressure used, at 50 g/cm^2^, 100 g/cm^2^, and 150 g/cm^2^; the first letter presents the type of specimen, where N indicates the control groups without CFA and F means that the fine aggregates had been replaced by CFA; the last numbers in the F specimens show the percentage of fine aggregates replaced by CFA, where 10 means 10 wt.% of the cement, 20 means 20%, and 30 indicates 30%.

The objective of this study was to determine the influence of the usage of CFA to replace partial fine aggregates and to determine the influence of pre-pressure on RCC engineering properties, including the physical properties, mechanical properties, durability and micro-structures. Table 4 shows the test items and referenced standards. In addition, three specimens of each mixture were tested and the average taken for each test. The standard deviation was controlled at less than 10% for the tested results. The specimens for XRD spectrum analysis were ground into powders and passed through a No. 200 sieve (75 μm). The XRD used involved Cu–Ka radiation at normal temperature, which was scanned from 2θ = 10°–80°.

## 3. Results

### 3.1. Compressive Strength

**Figure 2:**
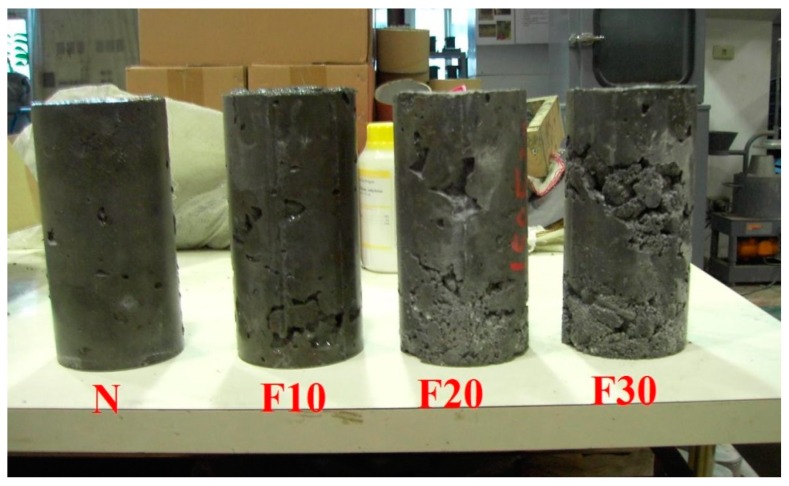
Appearance of roller-compacted concrete (RCC) specimens containing various amounts of CFA.

**Figure 3:**
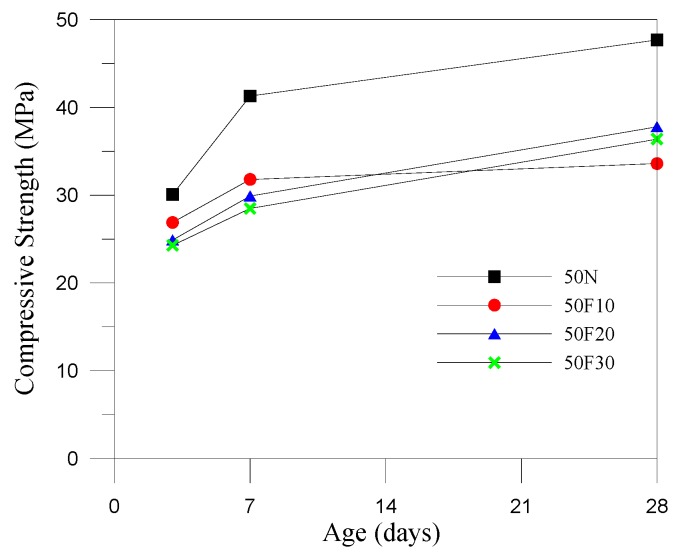
Compressive strength development curves (pre-pressure = 50 g/cm^2^).

**Figure 4:**
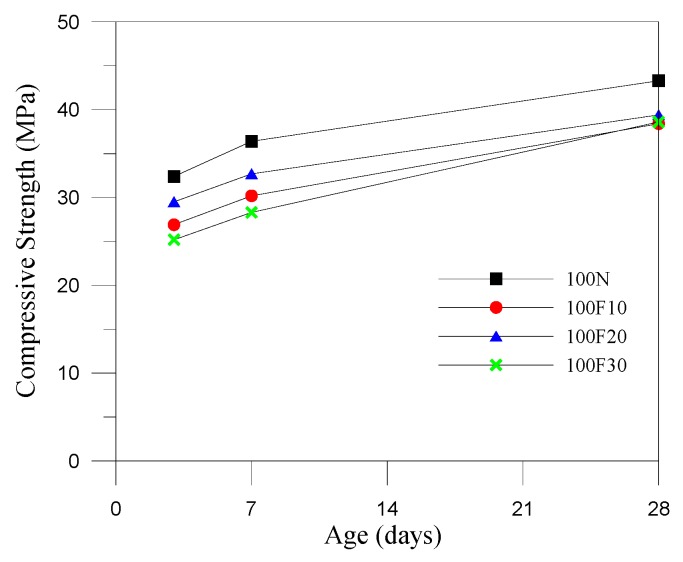
Compressive strength development curves (pre-pressure = 100 g/cm^2^).

**Figure 5:**
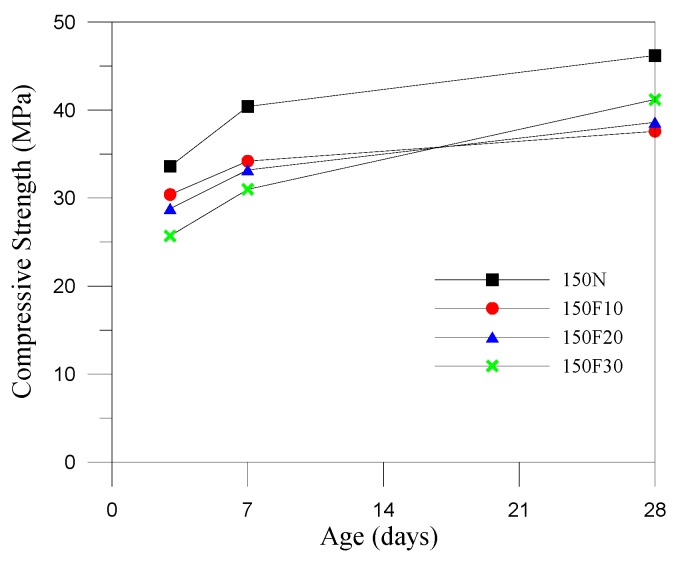
Compressive strength development curves (pre-pressure = 150 g/cm^2^).

Generally, the compressive strength of the fully compacted and vibrated RCC specimens is significantly higher than its original design strength at 28 days due to the lower water demand and higher coarse aggregate content. RCC mixtures must be dry enough to support the weight of a vibratory roller. Thus, we revised the amounts of water in the mix designs by controlling the vibrating-compacted (VC) value, which equaled 60 ± 20 s in the control groups. The consistency of RCC was examined by VC using the Vebe test, and the test results revealed that the water demand increases with the increase of fine aggregates replaced by CFA. This was an indirect result of the increase in water–binder ratios, which reduced the compressive strength of the specimens at 28 days. The design strength of the 50 N specimens (control specimens) was 35.0 MPa at 28 days, and the actual compressive strength of the 50 N specimens reached 47.7 MPa at 28 days, showing a significant increase. Thus, CFA can be considered a hydraulic material, as replacing the fine aggregates using CFA (at weight percentages of 0%, 10%, 20%, and 30%) can change the workability and setting time of the RCC. However, the specimens containing CFA showed a lower compressive strength compared with the control specimen; this may be because the compressive strength of CFA itself was not as high as that of the original fine aggregates. This may be due to the higher demand of water for higher CFA replacement in RCC, and several pores were observed in the surface of the RCC specimens (as shown in Figure 2). For strength development, the lower reactivity of the small particles of CFA (the particles passed through a No. 200 sieve (75 μm) amount to roughly 86% of the total) may be due to a cementitious property, and this was useful to aid in strength development at a later stage. Only specimen 50F10 did not meet the required standard, and the 10% replacement resulted in sparsely distributed particles and insufficient bonding properties. Figure 3, Figure 4 and Figure 5 show that CFA was not particularly beneficial for the early strength of the specimens, but resulted in a significant improvement in later strength. With the increase of pre-pressure (50 g/cm^2^, 100 g/cm^2^, and 150 g/cm^2^), the compressive strength of RCC containing CFA was slightly improved. However, the impact was less prominent in the control groups at 28 days, possibly because ASTM C1176 recommended limiting the pre-pressure of 50 g/cm^2^, and applying additional pressure can easily lead to bleeding, resulting in reduced strength. The 150F20 specimens had the largest compressive strength at 41.2 MPa.

### 3.2. Flexural Strength

**Figure 6:**
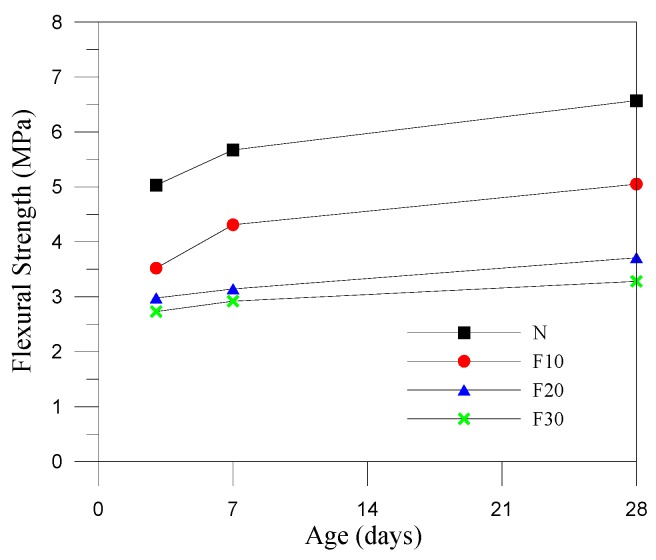
Flexural strength development of the specimens.

Figure 6 displays the results of the flexural strength test under a constant pre-pressure of 28 g/cm^2^. It was revealed that the increase of fine aggregates replaced by CFA increased with the water–cement ratio, and the testing trend is similar to that of the results of compressive strength. Nevertheless, the flexural strength of the specimens at 28 days remained within the recommended range from 3.49 MPa to 6.89 MPa. Only specimen F30 did not attain the required level of strength. As shown in Figure 6, increasing the aggregate replaced by CFA decreased the flexural strength of the RCC, which reached half its value for the N specimens at 28 days. However, once the CFA content exceeded 20%, this hindered the later development in flexural strength.

### 3.3. Absorption

The absorption results of the specimens at the age of 28 days are shown in Table 5. It can be seen that the absorption increased along with the increase of fine aggregates replaced by CFA. The likely explanation for this is that the absorption of CFA particles is higher than that of the fine aggregates. This is due to the porous and irregular surface of CFA; in addition, replacing fine aggregates with CFA reduced the workability, which increased the difficulty of specimen compaction and thereby increased the porosity (as shown in Figure 2). As shown in Table 5, the absorption did not decline when the CFA replacement increased from 0% to 20% and the pre-pressure increased from 50 g/cm^2^ to 150 g/cm^2^. It is speculated that the workability reduction effects of CFA outweighed the compacting effects of the pre-pressure. While the workability reduction was still an issue at a CFA content of 30%, the increase in pastes meant that more pores could be filled with pastes as the pre-pressure was applied, thereby increasing the compactness and reducing the absorption capacity.

### 3.4. Setting Time

The results are shown in Table 6. As can be seen, replacing the fine aggregates with CFA could shorten the initial setting time by 30% to 60% and shortened the final setting time by 16% to 20%. However, once the CFA content reached 30%, the F30 specimen could not reach the 3.5 and 27.6 MPa of the penetration resistance for the initial and final setting time, respectively. It speculated that this is due to the higher content of CaSO_4_ in CFA. Increasing the amount of CFA could result in more sulfate ions being imparted to the solution and lead to resistant ettringite formation. When the CFA percentage rose, the setting times were also extended [26]; this indicated that the increase of CFA content led to an increase in the water demand of RCC and a significant reduction in the setting time. As a result, using CFA in RCC makes it easy to change the setting time, which in turn affects workability.

### 3.5. Unit Weight

RCC has poor grading and is more compact than ordinary concrete. Its unit weight is generally higher than that of conventional concrete by 1% to 3%. Table 7 presents the unit weight results of the specimens. It can be seen that the unit weight decreases as the increase of fine aggregates replaced by CFA and increases as the pre-pressure is applied. Theoretically, the inclusion of CFA in RCC should increase the unit weight because of its smaller specific gravity; however, due to the higher CaO content in CFA, the hydration reaction is much quicker than that of cement, which increases the difficulty of specimen compaction and results in greater porosity. Consequently, the unit weight decreases along with the increase of the replacement of fine aggregate by CFA. Increasing the pre-pressure enhances workability and presents better compacting effects, which help to increase the unit weight.

### 3.6. Sulfate Resistance

This test was performed according to ASTM C88 to simulate the conditions of RCC in environments rich in sodium sulfate for prolonged periods and examine the sulfate resistance of the various specimens. As shown by the results in Table 8, the control specimens were reduced in compressive strength due to the corrosion suffered, whereas the specimens containing CFA had a greater compressive strength than before the test.

However, changing the pre-pressure does not significantly improve the sulfate resistance. The ettringite produced during the test generally resulted in volume expansion and damage. However, replacing the fine aggregates with CFA increased porosity and resulted in the expansion of the ettringite filled in the pores, thereby increasing the compactness of the specimen. This indicated that the CaO and Ca(OH)_2_ in the CFA reacted with the H_2_SO_4_ in the solution of sodium sulfate to produce even more CaSO_4_·2H_2_O, which filled in the pores and further enhanced the compressive strength of the specimens [1,16]. The hydration reactions can be written as follows [27]:

CaO + H_2_O → Ca(OH)_2_ (1)

2Ca(OH)_2_ + H_2_SO_4_ → CaSO_4_ + 2H_2_O (2)

### 3.7. SEM Observation

**Figure 7:**
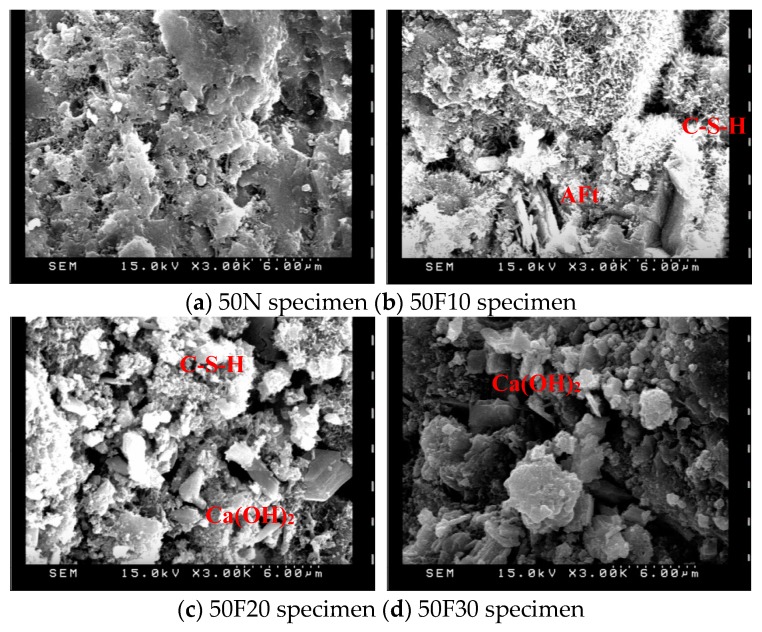
SEM observations (pre-pressure = 50 g/cm^2^, ×3000); (**a**) 50N specimen (**b**) 50F10 specimen; (**c**) 50F20 specimen (**d**) 50F30 specimen.

**Figure 8:**
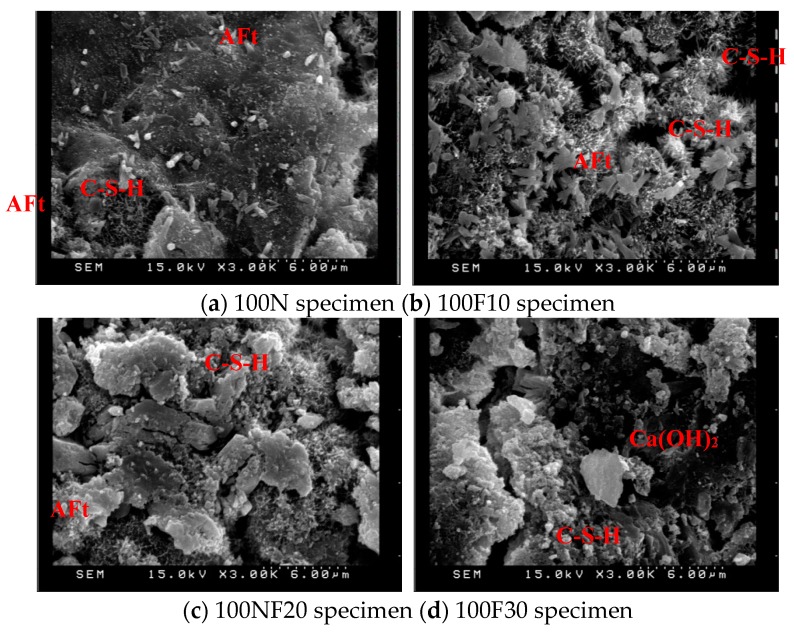
SEM observations (pre-pressure = 100 g/cm^2^, ×3000).

**Figure 9:**
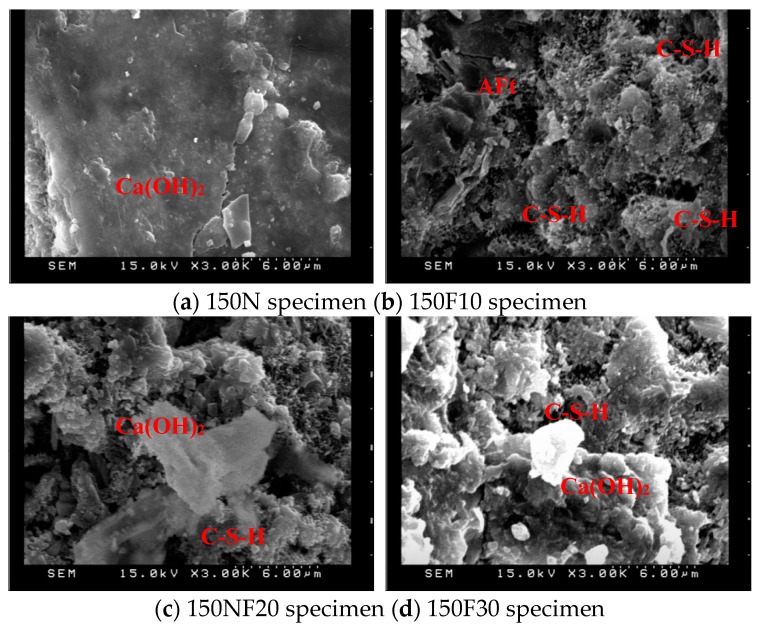
SEM observations (pre-pressure = 150 g/cm^2^, ×3000).

**Figure 10:**
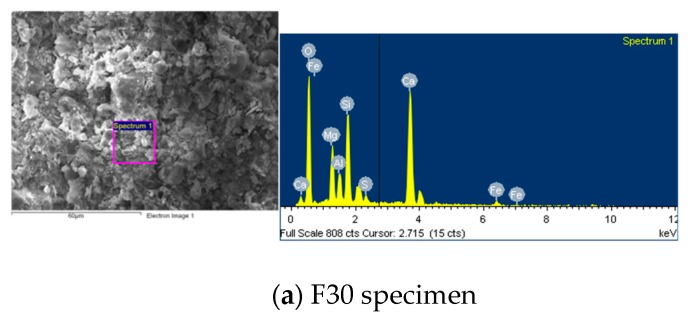

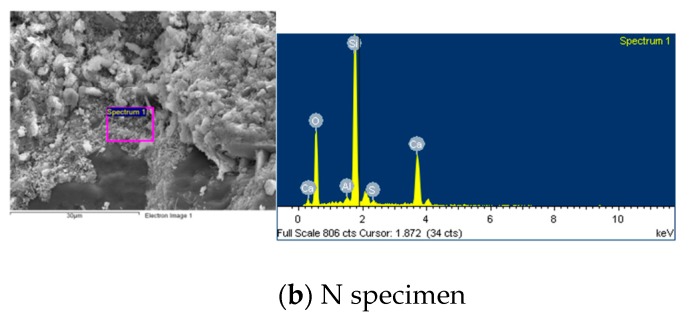
SEM photos with energy dispersive X-ray spectroscopy (EDS) analysis.

The products of the hydration reaction between the water and cement in concrete generally include spiky spheres of C–S–H gel, hexagonal or irregularly-shaped plates of Ca(OH)_2_, hexagonal plates or irregular rosettes of mono sulfoaluminate, and elongated tubular needles—sometimes intertwined—of ettringite (AFt). The formation of AFt and C–S–H filled up the pores and enhanced the compaction of the specimens [28]. After 28 days of curing, samples of 10 × 10 × 3 mm^3^ were observed with SEM, the results of which are displayed in Figure 7, Figure 8 and Figure 9. As can be seen, increasing the amount of fine aggregate replaced with CFA increases the porosity. At the same time, however, increasing the pre-pressure also increases the compactness, thereby verifying the results of the compressive strength test. The small particles of CFA may be provided with a cementitious property, and this was useful to aid in strength development at a later stage. It was confirmed that the CaO contained in CFA is converted into Ca(OH)_2_ by hydration and used for the formation of ettringite and calcium silicate hydrate [29]. A higher content of free calcium oxide from CFA is beneficial to the generation of more ettringite and calcium silicate hydrate. The results of the SEM photos containing EDS analysis are shown in Figure 10. These indicated that the Ca(OH)_2_ content in the specimens increased along with the increase of CFA. The N30 specimens had lower amounts of SiO_2_, and this indicated that the Ca(OH)_2_ and f-CaO in CFA reacted with SiO_2_, leading to the formation of calcium silicate hydrate.

### 3.8. XRD Spectrum Analysis

**Figure 11:**
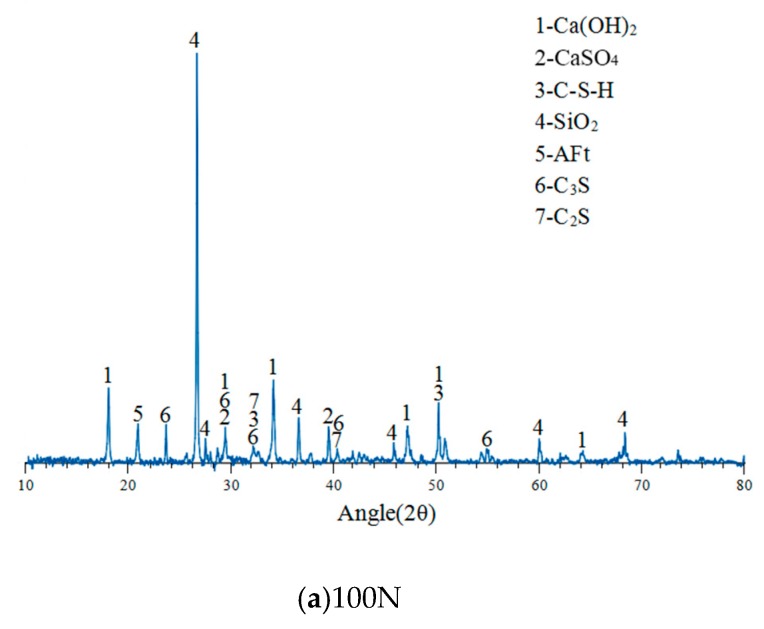

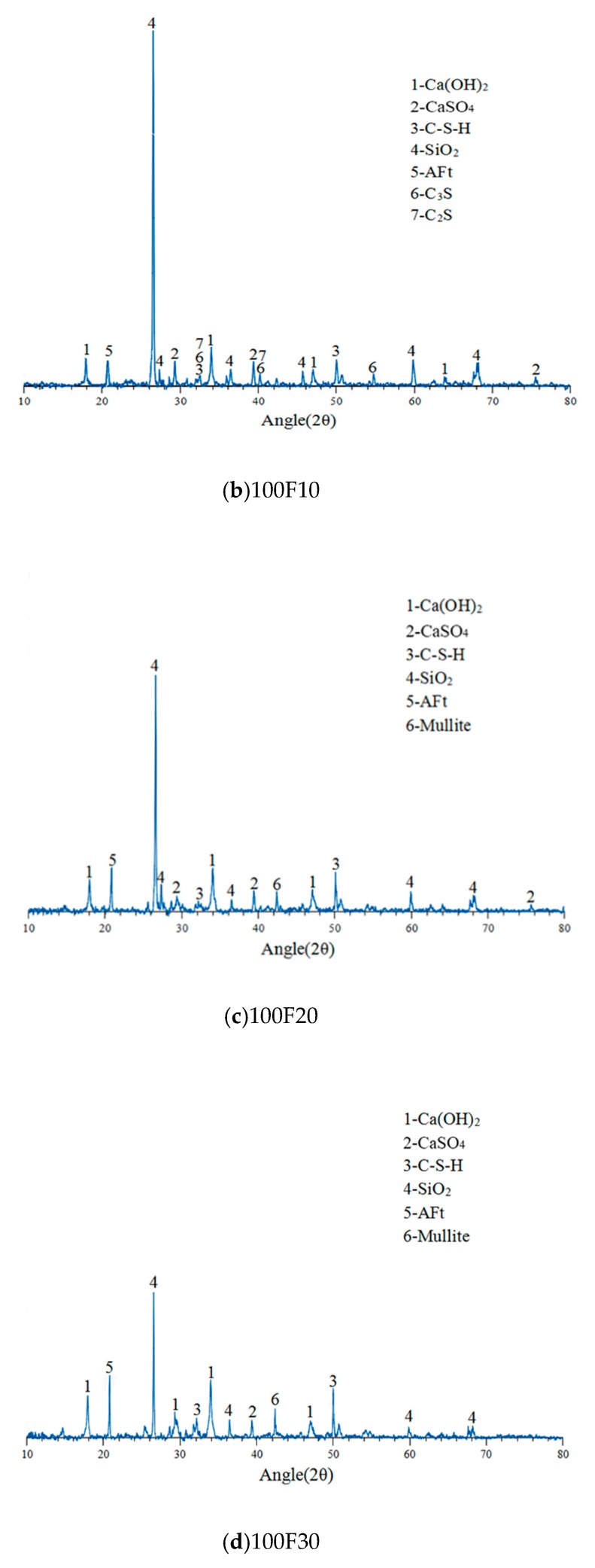
XRD spectrum for various specimens.

We used XRD analysis to examine the powder of specimens cured for 28 days, and the results can be seen to maintain the trend of the hydration. The results are as shown in Figure 11a,b. The main hydration products of the specimens included Ca(OH)_2_, CaSO_4_, SiO_2_, Aft, and C–S–H gel. With the increase of the dosage of CFA, the intensity of the diffraction peaks of SiO_2_, C_2_S, and C_3_S in the specimens gradually decreased. When the CFA replacement exceeded 10%, fewer C_2_S and C_3_S diffraction peaks were seen. In addition, the C–S–H diffraction peak was gradually increased when the CFA replacement was 30%. As can be seen, as the amount of fine aggregate replaced by CFA increases, the Ca(OH)_2_ content in the specimens increases and the CaSO_4_ content decreases. This may be because the CaO in the CFA undergoes a hydration reaction during the solidification and produces Ca(OH)_2_, which is in agreement with the results of SEM observations.

When the CFA replacement exceeds 20%, not only was more CSH generated, but also more Ca(OH)_2_ and ettringite crystals were produced. The expansion and destruction effect produced by ettringite crystals and Ca(OH)_2_ was higher than that of the blending effect produced by C–S–H gel and eventually led to a reduction in compressive strength at 28 days, which is consistent with the results of compressive strength. Previous studies indicated that quartz (SiO_2_) in CFA reacted with lime (CaO) and calcium hydroxide (Ca(OH)_2_), leading to the formation of anhydrite (CaSO_4_) [16,27,30]. It can be speculated that a small part of CFA particles acted as cementitious materials and most CFA particles acted as fillers in RCC based on the results of XRD and SEM.

## 4. Conclusions

1. The inclusion of CFA as a substitute material for fine aggregates has a significant influence on the engineering properties of RCC. The compressive strength has no obvious improvement as a result of the increase of fine aggregates replaced by CFA due to the increase of water demand, but this significantly enhances later strength due to the additional hydration.

2. The results of the flexural strength test indicate that the strength at the age of 28 days can achieve the recommended range (3.49 MPa to 6.89 MPa). Flexural strength significantly decreased with an increase of fine aggregate replaced by CFA.

3. The inclusion of CFA can reduce the setting time, absorption and unit weight, and increase the sulfate resistance in RCC.

4. Pre-pressure is a key factor, and the increase in pre-pressure helped to reduce the absorption, flexural strength and setting time, as well as to increase the unit weight and compressive strength.

5. The sulfate resistance test results show that the increase of fine aggregates replaced by CFA enhanced the sulfate resistance of RCC. In contrast, the increase of the pre-pressure did not have significant effects. Furthermore, as the amount of CaSO_4_·2H_2_O in the specimens increased during the test, the compactness and compressive strength of the specimens also increased.

6. SEM observations showed that the increase of fine aggregates replaced by CFA improved the compactness and crystal morphology of the C–S–H gel in the specimens.

7. The XRD spectrum analysis indicated that the increase of fine aggregates replaced by CFA increased the content of Ca(OH)_2_ and decreased the content of CaSO_4_ in the specimens.

8. It is recommended to use an RCC containing 10% CFA as a replacement for fine aggregates under a roller compaction pressure of 100 g/cm^2^.

## Figures and Tables

**Table 1:** Heavy metal content of CFA.

Metal	Test Results (ppm)	Control Standard (ppm)
Chromium (Cr)	4.43	250
Nickel (Ni)	191.97	200
Copper (Cu)	1.93	400
Zinc (Zn)	-	2000
Arsenic (As)	-	60
Cadmium (Cd)	-	20
Lead (Pd)	2.02	2000

**Table 2:** Chemical constituents of CFA.

Chemical Constituent	Percentage (wt.%)
Loss on ignition (LOI)	7.83
Silicon dioxide (SiO_2_)	3.72
Aluminum oxide (Al_2_O_3_)	0.55
Ferric oxide (Fe_2_O_3_)	0.57
Calcium oxide (CaO)	55.84
Magnesium oxide (MgO)	1.62
Sulfur trioxide (SO_3_)	29.09
Potassium oxide (K_2_O)	0.34
Sodium oxide (Na_2_O)	0.10
Strontium oxide (SrO)	0.1

**Table 3:** Mix designs of specimens containing different amounts of CFA (kg/m^3^).

Mix No.	Water	Cement	Fine Aggregates	Coarse Aggregates	CFA
50N	134.4	322.0	717.4	1245	0.0
50F10			645.6		71.8
50F20			573.9		143.5
50F30			502.2		215.2
100N			717.4		0.0
100F10			645.6		71.8
100F20			573.9		143.5
100F30			502.2		215.2
150N			717.4		0.0
150F10			645.6		71.8
150F20			573.9		143.5
150F30			502.2		215.2

**Table 4:** Test items and referenced standards. SEM: scanning electron microscopy.

Test Type	Test Method	Specimen Dimensions (mm) and Types	Referenced Standards
Fresh properties	Setting time test	150 × 150 × 150 (mortar)	ASTM C403
	Unit weight test	φ 100 × 200 (concrete)	-
	Vebe test	-	ASTM C1170
Mechanical properties	Compressive strength test	φ 100 × 200 (concrete)	ASTM C39
	Flexural strength test	150 × 150 × 530 (concrete)	ASTM C78
Durability	Absorption test	φ 100 × 200 (concrete)	ASTM C642
	Sulfate resistance test	φ 100 × 200 (concrete)	ASTM C88
Characterization	SEM observation	10 × 10 × 3 (mortar)	ASTM C1723
	XRD spectrum analysis	Powders	ASTM C1365

**Table 5:** Absorption of specimens.

Mix No.	Absorption (%)	Difference from the Control Group (%)
50N	3.2	0
50F10	4.3	1.1
50F20	4.8	1.6
50F30	5.7	2.5
100N	3.7	0
100F10	4.6	0.9
100F20	5.0	1.3
100F30	5.2	1.5
150N	3.5	0
150F10	4.1	0.6
150F20	5.0	1.5
150F30	5.2	1.7

**Table 6:** Setting times of specimens.

Mix No.	N	F10	F20	F30
Initial setting time (min)	80	30	55	-
Final setting time (min)	150	120	125	-

**Table 7:** Unit weight of specimens.

Mix No.	Unit Weight (kg/cm^3^)
50N	2451.0
50F10	2438.3
50F20	2402.2
50F30	2381.0
100N	2474.3
100F10	2456.3
100F20	2408.5
100F30	2405.4
150N	2504.0
150F10	2474.3
150F20	2418.1
150F30	2413.9

**Table 8:** Compressive strength of specimens before and after sodium sulfate immersion.

Mix No.	Pre-Test Compressive Strength (MPa)	Post-Test Compressive Strength (MPa)	Variation in Strength (%)
50N	47.7	46.5	−2.5
50F10	33.6	44.3	31.8
50F20	37.8	42.2	11.6
50F30	36.4	39.6	8.8
100N	43.3	41.8	−3.5
100F10	38.4	47.6	24.0
100F20	39.4	41.3	4.8
100F30	38.6	42.9	11.1
150N	46.2	44.2	−4.3
150F10	37.6	43.3	15.2
150F20	38.6	49.8	29.0
150F30	47.7	46.3	12.4
